# Multiscale modeling of the spatial structure of stem cells in neuroblastoma patient-derived tumoroids reveals a critical role for a short-range diffusive process

**DOI:** 10.1371/journal.pcbi.1014137

**Published:** 2026-03-31

**Authors:** Thi Nhu Thao Nguyen, Catherine Koering, Elodie Vallin, Sandrine Gonin-Giraud, Laura Broutier, Samuel Bernard, Fabien Crauste, Olivier Gandrillon

**Affiliations:** 1 Université Paris Cité, CNRS, MAP5, F-75006 Paris, France; 2 LBMC, ENS de Lyon, CNRS, UMR 5239, Inserm, U1293, Universite Claude Bernard Lyon 1, 46 allee d’Italie F-69364 Lyon, France; 3 Inria, Paris, France; 4 Childhood Cancer & Cell States Team (C3 Team), LabEx DEVweCAN, Institut Convergence Plascan, Centre Léon Bérard, Centre de Recherche en Cancérologie de Lyon (CRCL), Université Claude Bernard Lyon 1, INSERM 1052, CNRS 5286, 69008 Lyon, France; 5 Univ Lyon, Université Lyon 1, CNRS UMR5208, Institut Camille Jordan, 43 Blvd du 11 Novembre 1918, Villeurbanne-Cedex F-69622, France; University of Utah, UNITED STATES OF AMERICA

## Abstract

Neuroblastomas are heterogeneous pediatric tumors of the sympathetic nervous system for which treatments are still limited. Fundamental and applied approaches have been enabled thanks to the generation of patient-derived tumoroids (PDT), ex vivo 3D structures used as avatars of the original tumor. We generated neuroblastoma PDT and quantified the spatial distribution of CD133^+^ cancer stem cells using immunohistochemistry. We observed that those cells tend to aggregate in the PDT. In order to better understand the set of rules needed for generating such structures, we implemented a multiscale agent-based neuroblastoma tumoroid model. Model rules specify single cell’s fate based on their intracellular content, which dynamically evolves according to a stochastic gene regulatory network. The state of this network can be modulated by cell-to-cell signaling through neighbor cell fate decisions and, possibly, spatial location. We first observed that in the absence of any spatial rules for inter-cellular interactions, no spatial structure emerged. The addition of simple rules (signaling by cell-to-cell contact or differential cell adhesion) only marginally improved the quantitative agreement to the experimental dataset. In sharp contrast, the addition of short-range pro-stem cell diffusive signaling among stem cells produced very realistic 3D PDT-like structures. This works highlights the power of our multiscale approach to discard too simplistic rules and to propose a minimal set of hypotheses required to reproduce qualitatively and quantitatively experimentally observed spatial structures. In the case of neuroblastoma-derived PDT, short-range spatial diffusion of stem-to-stem cell signaling proved to play a key role in successfully reconstructing the spatial structure.

## Author summary

Neuroblastomas, the most prevalent pediatric tumors of the nervous system in children under five, still have limited treatment options. Recent advancements allow the creation of tumoroids—three-dimensional structures derived from and recapitulating features of patient tumors—which are crucial for neuroblastoma research. We generated such tumoroids and noted a distinct spatial organization where cancer stem cells, key targets for treatment, cluster within tumors. A multi-scale computational model was developed to simulate tumoroids growth and cancer stem cell differentiation, based on gene activity related to stem and non-stem cell phenotypes. The model revealed that simple or absent inter-cellular interaction rules could not replicate the spatial organization of tumoroids. However, introducing a short-range, pro-stem cell diffusive signal produced realistic three-dimensional structures quantitatively similar to experimental observations. Our study highlights the effectiveness of a multiscale computational approach, enabling the creation of an in silico neuroblastoma tumoroids growth model and offering insights into the specific spatial structures observed experimentally, potentially leading to innovative treatments.

## Introduction

Neuroblastomas (NB) are pediatric tumors of the sympathetic nervous system derived from primitive neural crest cells. Although rare, neuroblastoma is the most frequent extracranial solid tumor in children, primarily occurring in those under five years old. It accounts for 8–10% of all childhood cancers and ranks as the third most common childhood malignancy [1, 2]. NB is a tumor with heterogeneous biological, morphological, genetic and clinical characteristics. NB patients have been stratified into low-, intermediate-, and high-risk groups. Low-risk patients may show spontaneous regression or respond favorably to treatment, leading to markedly superior survival. On the other hand, the current treatment regimens are insufficient for patients with high-risk NB [3]. Only few actionable mutations have been identified including ALK [4] and the molecular regulatory network surrounding MYCN [5], a gene amplified in ~25% of neuroblastoma patients [6] and affecting stemness of neuroblastoma cells. Very few drugs have been validated clinically [7], therefore stressing the need for more innovative approaches.

The recent possibility to generate *ex vivo* relevant 3D structures that recapitulate at least part of the histological architecture typical of NB has paved the way for both fundamental and applied approaches on Patients-Derived Tumoroïds (PDT) from children suffering from NB [8, 9]. To characterize neuroblastoma spatial structure, we developed PDT cultures from a NB tumor and used immunohistochemistry (IHC) to assess its neuroblastoma nature. We observed a characteristic spatial positioning of CD133^+^ cancer stem cells (CSC) that tend to be clustered around the center of the PDT. CSCs have been defined by analogy with normal stem cells as a subpopulation of (mostly rare) cancer cells thought to be responsible for tumor self-renewal and deregulated differentiation into various cancer types [10]. To better understand the mechanisms at work behind structure formation, we implemented a computational model of PDT growth and assessed the ability of this model to investigate and reproduce growth and differentiation spatial patterns.

Ideally the computational modeling framework should be able to accommodate the fact that cell decision making is a multiscale dynamical process, which associates an intracellular genome-based gene regulatory network (GRN), which is “stochastic at every level of organization” [11], a cellular behavior, and a tissue-level organization. Each level can both influence and be influenced by other levels. For example, it has been shown that tissue organization exerts stabilizing constraints against noise on the cellular level [12]. The cellular behavior emerges from the GRN dynamics but the molecular level can also be influenced by cellular level constraints like the cell metabolic state [13].

There is a large body of literature on stem cell modeling (whether cancerous or not) and cancer cells (whether stem or not) but only a handful of models have been used to explore the CSC dynamics within tumors [14–22].

While some studies rely upon ordinary differential equations to model CSC dynamics (and therefore neglect spatial aspects; [22]), most of these studies rely upon the use of agent-based models (ABM). All of those ABM are built on a 2D lattice grid, except for [15] which uses a 2D continuous space (implemented in NetLogo). Although none of the models simulate GRN, some studies [15, 20] can be described as multiscale models: Biava et al. [15] models how molecular factors (peptides, microRNAs) influence tumor cell behavior, connecting the molecular and cellular levels through probabilistic rules, while Fotinos et al. [20] links cell-level behavior (division, differentiation) to tissue-level patterns of CSC organization.

Additionally, while several models [14, 17–19, 21] are purely conceptual and theory-driven, demonstrating how tumor-like behavior can arise from basic cell-level rules, others (notably [15] and [20]) incorporate quantitative validation using experimental data, such as fluorescence imaging, cell marker intensity, and graph-based spatial statistics.

Mathematical and computational models of neuroblastoma remain relatively scarce, especially compared to those for other cancer types and the extensive literature on stem cell modeling. Most existing mathematical and computational models focus on subcellular or molecular mechanisms [23–26] or treatment schedule optimization by modeling clonal evolution [27].

Agent-based models have been widely used in tumor modeling to represent individual cell behavior and cell-cell microenvironment interactions in 2D systems [28, 29], as well as tumor spheroid growth in 3D for generic cancer type [30]. Only a small number of recent studies have proposed 3D computational models of neuroblastoma tumor growth and evolution, including spatially explicit 3D frameworks. Noticeably, Boreau et al. [31] introduced a computational, cloud-based model developed by the PRIMAGE consortium in order to help clinicians investigate the disease progression and optimize patients treatments. Wertheim et al. [32] proposed a continuous automaton model describing the growth of a tumor in a 3D domain, including several features of interest such as the extra-cellular environment or cell mutations, and investigated the progression and response to various treatments of MYCN-amplified clones. As stated by the authors, a clear limitation of both approaches is that neither their model include dynamic models of the signalling pathways and gene regulatory networks [31] nor do their static representation allows for a dynamical modelling inside the agent within one time step [32].

Reviewed studies offer valuable conceptual and computational insights into CSC dynamics and tumor growth, but they share several notable limitations. Most of these models are confined to 2D lattice-based environments, which restricts spatial realism and may oversimplify the complexity of *in vivo* tumor structure. GRN and intracellular signaling pathways are absent, limiting the models’ ability to capture the molecular mechanisms underlying cell fate decisions. This might prove especially limiting when the model’s output might be confronted to spatial transcriptomics data [33].

To overcome these limitations, we developed Simuscale [34, 35], a multiscale agent-based modeling framework that incorporates a stochastic GRN in each cell, the state of which can be influenced by cell-to-cell signaling. The resulting model is truly multiscale in the sense that it harbours at least two nested organization scales and interactions between and among scales. This results in a circular causality scheme [36] that can not be easily apprehended by more traditional modeling schemes.

Simuscale enables simulations from molecular to population scales, while considering both spatial movement and signaling dynamics, whether through direct cell-to-cell (local) contact or through long-range diffusion.

The model introduced in this paper consists of 15 initiating stem cells, endowed with a GRN consisting of a simple toggle-switch between two proteins controlling cell fate, and regulating a protein controlling cell proliferation. This GRN was modelled as a piecewise deterministic Markov process operating under the bursty regime [37], which we previously demonstrated to be able to reproduce realistic single-cell transcriptomics data [38]. In the absence of any spatial rule, the model proved insufficient to reproduce qualitatively or quantitatively the observed IHC images. The addition of simple spatial rules, like stem-to-stem cell contact and cell movement, only marginally improved the simulation outcomes. The key to successful reconstruction of the observed spatial structure of PDT proved to be a stem-to-stem short range diffusive signaling process.

## Results

### Immunohistochemistry

We analyzed the 10 IHC images obtained from five PDT from the same culture (see section Histological analyses). S1 Fig shows that 100% of the cells displayed a strong PHOX2B signal, thereby establishing their NB identity. Additionally, the amount and intensity of staining for the three antigens was fully conserved between the Patient-derived xenograft and the PDT (and when available the original tumor), demonstrating that culture conditions did not induced any significant drift in cell identity. Finally, the expression of CD56 and synaptophysin (thereafter abbreviated SYP) was clearly less homogeneous than the expression of PHOX2B, with around 50% of the cells stained positive. This is in line with the previous description that PDT can recapitulate at least partly NB tissue heterogeneity.

We then focused on the spatial structure observed in IHC images, based on the expression of CD133, defining a stem cell subpopulation, and SYP, defining a differentiated cell subpopulation.

#### Spatial distribution of stem cells.

**Fig 1:**
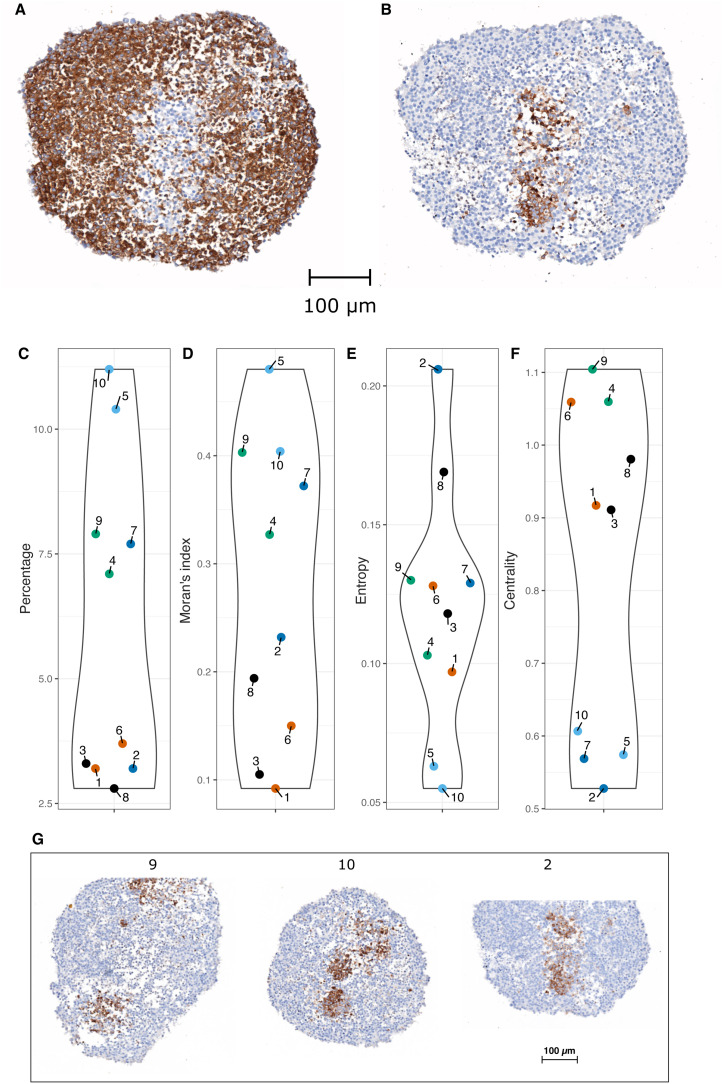
Stem cell spatial distribution. A–B. Synaptophysin labelling (A) and CD133 labelling (B) are shown in two consecutive µm-thick tissue sections of the same PDT. A cell appears brown if it expresses the protein; otherwise, it colors blue. C. Percentage. D. Moran’s Index. E. Entropy. F. Centrality. Points labeled 1 to 10 correspond to the PDT images T1 to T10. The same color is used for the values computed on two sections from the same PDT. G. IHC images: CD133 labelling is shown for three PDT (T9, T10 and T2), a cell appears brown if it expresses the CD133 protein.

Fig 1 presents the quantitative characterization of the spatial distribution of stem cells in the IHC images.

Fig 1A and 1B represent IHC image T7 of PDT, showing the expression of SYP and CD133, respectively, in two different sections in close proximity. It is immediately apparent that the expression of those two proteins is mutually exclusive, suggesting the existence of a toggle switch mechanism between these two genes.

We used four statistical indices (introduced in section Statistical methods) to quantitatively characterize the spatial distribution of CD133^+^ cells. Stem cell percentage across the 10 IHC images (Fig 1C) ranges from 2.8% to 11.2%. It is apparent that there is a relatively large variation among PDT (ranging from low values for T3 and T8 to high values for T5 and T10). Two sections arising from the same PDT display very close percentages, except for T2 and T7.

Moran’s *I* (Fig 1D) ranges from 0.09, which is close to a random distribution, to 0.48, which indicates a clear spatial clustering. Here again, some section-to-section variability from the same PDT can be observed, again more pronounced for T2 and T7.

Entropy index values (Fig 1E) range from 0.05 to 0.21. This index provides additional insights into stem cell clustering, especially in cases where Moran’s *I* values are similar, by quantifying the shape of the clustering. The more elongated the cluster is, the higher the entropy. For instance, IHC images T9 and T10 (Fig 1G) share the same Moran’s *I* value, yet their entropy values differ. This difference arises because image T9 contains two well separated stem cell clusters, whereas T10 displays more compact clusters, leading to more dispersed cell-to-cell distances.

The centrality index of stem cells (Fig 1F) shows values ranging from 0.53 to 1.1. Low centrality values indicate that stem cells are more concentrated near the center of the image, as illustrated in Fig 1E for image T2, while high centrality values highlight stem cells located away from the center of the image (see T9, on Fig 1G).

#### Variance statistics for spatial dispersion of stem cells.

One key question is how to ascertain the statistical significance of an empirical deviation from purely random spatial configurations. In the absence of a proper analytical expression, the significance is usually obtained by simulating many random permutation configurations by Monte Carlo simulations. When applied to Moran’s *I*, this requires a huge amount of permutations (approximately 10^7^), and therefore a very large computation time.

Jensen and Michel [39] provide an analytic formula to compute the 95% confidence interval of so-called intra-coefficient $a_{SS}$ in the case of purely random distributions of points, and therefore alleviates the need for such permutations experiments. This allows testing *H*_0_, the hypothesis that the spatial locations of stem cells are purely random, the alternative hypothesis *H*_1_ being that cells are not randomly distributed. In this case, one has to examine whether $a_{SS}$ stays above the reference value (i.e., 1) in which case one can conclude that cells are significantly clustered, or below the reference value in which case one can conclude that cells are significantly dispersed.

**Fig 2:**
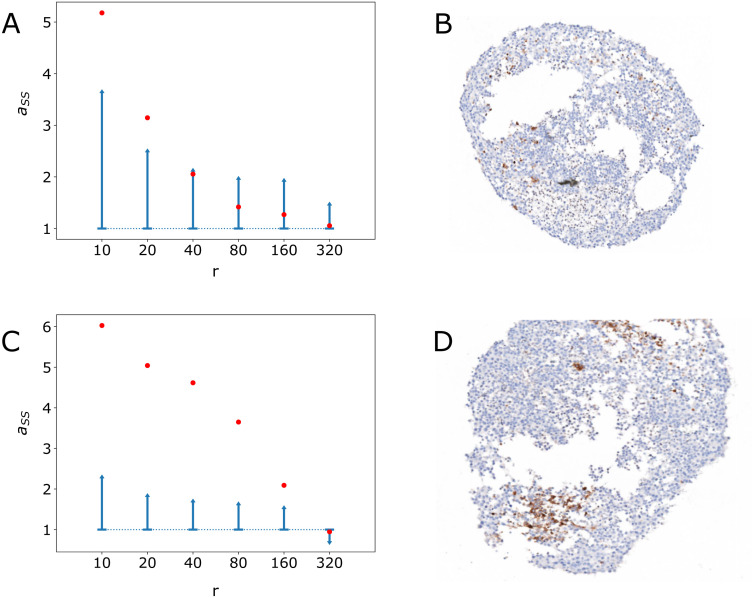
Plots of the intra-coefficient $a_{SS}$ for stem cells with respect to the radius *r.* A and C: in red is shown the value of the intra-coefficient computed on IHC image T1 (A) and T4 (C), and in blue is shown the upper half of the 95% confidence interval, as a function of the radius (*r*, µm). The actual images of T1 and T4 are shown in B and D, respectively.

Fig 2 presents the intra-coefficient $a_{SS}$ and its confidence interval under *H*_0_ (see section Statistical methods) across different values of the radius *r* for two IHC images (T1 and T4).

The intra-coefficient $a_{SS}$ remained well above the confidence interval until a certain radius (40 µm for image T1 and 320 µm for image T4) is attained. This allows to reject *H*_0_ and shows that stem cells are significantly clustered up until a certain characteristic radius value. In particular, for small radius, stem cells are always clustered. For larger radius values, $a_{SS}$ falls within the confidence interval, *H*_0_ can not be rejected, and therefore stem cells spatial locations tend to follow a random pattern.

The value of *r* at which *H*_0_ gets rejected is much higher for T4 (Fig 2C) than for T1 (Fig 2A), demonstrating that T4 displays larger clusters of stem cells than T1. It is however noteworthy that the T1 clusters that were characterized by a low Moran’s *I* nevertheless proved to be significantly different from a purely random spatial distribution. All 10 PDT images displayed significant clustering above the expected variance under *H*_0_ (the smallest radius value among all images was found for image T1 and equals 40 µm).

### Computational model

#### Cell fate.

**Fig 3:**
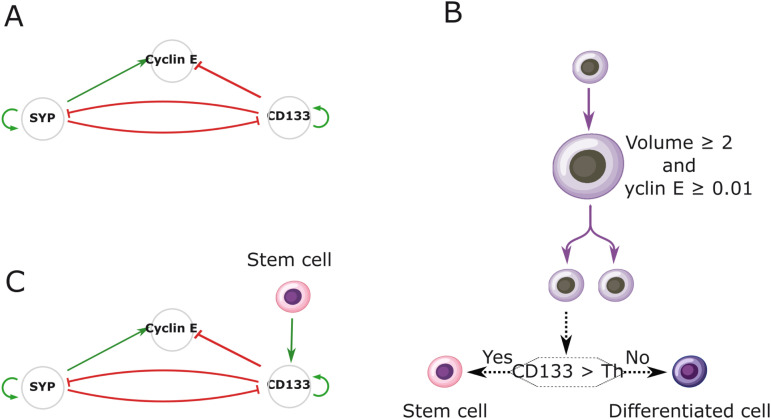
Model of cell dynamics and gene interactions. A. The first GRN implemented. It is formed of three genes, CD133, SYP, and Cyclin E, where CD133 and SYP interact through a toggle switch, and Cyclin E drives the proliferation. B. Rules for proliferation and differentiation at the cellular level. Cells grow and then divide depending on their volume and the level of expression of the Cyclin E protein. The differentiation decision is taken in each daughter cell following division. The differentiation fate depends on the comparison between the CD133 protein concentration and a given threshold, Th. C. Modified GRN, where stem cell signaling influences the burst frequency of gene CD133.

We consider a dynamic model of gene activity, where molecular values are governed by a GRN that includes a toggle switch between CD133 and SYP genes (Fig 3A). These two genes allow to define two cell subpopulations: stem cells (CD133^+^) and differentiated cells (SYP^+^), as obtained experimentally (section Immunohistochemistry). The GRN also incorporates the Cyclin E gene, which is used as a marker of proliferation. In this network, CD133 inhibits the burst frequency of Cyclin E, while SYP activates it, reflecting the fact that stem cells divide more slowly than differentiated cells [40]. The interaction matrix characterizing gene dynamics is provided in Table 1.

**Table 1:** Parameter values of the gene interaction matrix.

$\theta_{\text{CD133},\text{CD133}}$	15	$\theta_{\text{SYP},\text{SYP}}$	15	$\theta_{\text{CyclinE},\text{CyclinE}}$	0
$\theta_{\text{CD133},\text{SYP}}$	−40	$\theta_{\text{SYP},\text{CD133}}$	−40	$\theta_{\text{CyclinE},\text{CD133}}$	0
$\theta_{\text{CD133},\text{CyclinE}}$	−2	$\theta_{\text{SYP},\text{CyclinE}}$	250	$\theta_{\text{Cyclin E},\text{SYP}}$	0

We then define a model of stem and differentiated cell dynamics in which cell proliferation and differentiation dynamics are controled by the GRN (Fig 3B). Each cell has a constant volume growth rate. When its volume has at least doubled (the minimum volume is set to 1), the cell divides if the protein concentration of Cyclin E gene reaches the proliferation threshold, set to 0.01. During division, the two daughter cells inherit half of the molecular contents from their mother cell. Cell differentiation is assessed at each division: if the CD133 protein level of a daughter cell exceeds the differentiation threshold, the cell becomes or remains a stem cell; otherwise, it is deemed to be a differentiated cell.

Additionally, we propose a refined network that incorporates signaling (Fig 3C). Such signaling occurs either when a stem cell comes into contact with another stem cell, or through the diffusion of a stem cell-based signal (see section Signaling). This signaling activates CD133 by increasing its bursting frequency. As a consequence, signaling increases the likelihood of neighbouring cells to remain or become stem cells.

#### Spatial structure, differentiation threshold and cell-cell contact signaling.

Our objective was to generate model’s outputs ranging, for each of the four statistical indexes (see section Statistical methods), within the experimentally observed values.

**Fig 4:**
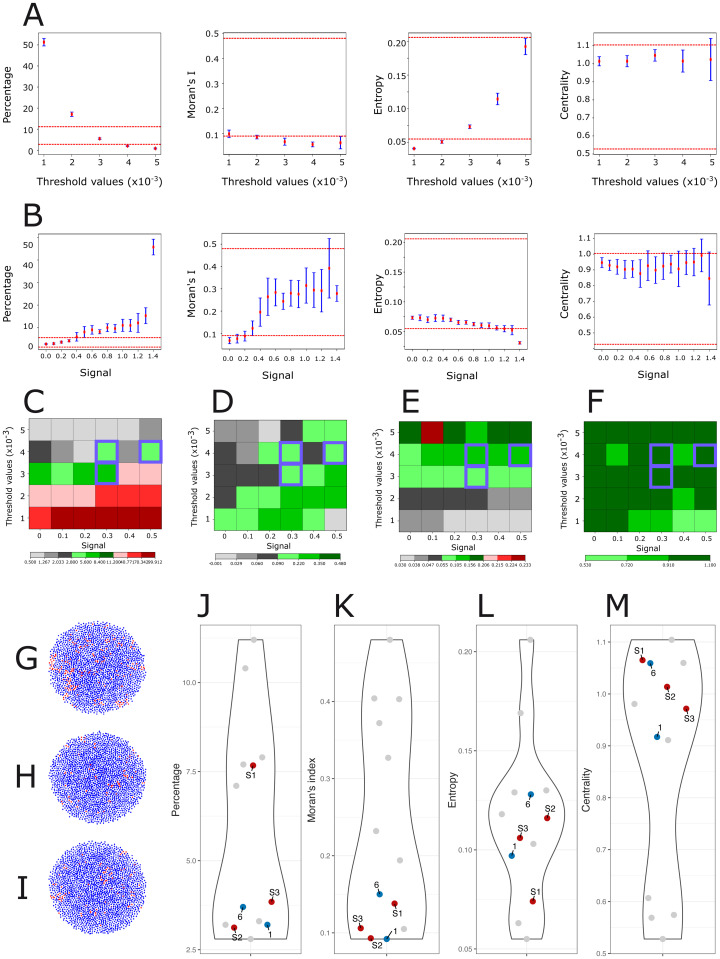
Assessing the role of differentiation threshold and cell-cell contact signaling. A. The behavior of our four indices for different differentiation threshold values. A central section was taken along the Z-axis to generate a 2D image, resembling IHC images. The mean values (red points) and standard deviations (blue lines) were then calculated. Two dashed red lines indicate the minimum and maximum values for each index based on experimental results (see Fig 1). From left to right is shown the stem cell percentage, the Moran’s *I*, the entropy index and the centrality index. B. The behavior of our four indices for different cell-cell signaling intensity, using a differentiation threshold value = 0.003. C–F. The four indices across different levels of threshold differentiation and signaling intensity (C: Percentage; D: Moran’s *I*; E. Entropy; F: Centrality). Each square represents the simulated value for a specific combination of threshold differentiation (Y-axis) and signaling strength (X-axis). The color bar is from now on used to categorize the values of each index, where green indicates values within the range of minimum to maximum obtained from the experimental results (i.e., acceptable values); black indicates values below the experimental minimum; red indicates values above the experimental maximum. Each of these colors has three levels of gradation, where darker shades represent greater values. Violet squares indicate values for which all four indices are simultaneously acceptable. G–I. 2D cross section through the center of the simulated PDT S1, S2 and S3. Stem cells are shown in red and differentiated cells in blue. J–M. Comparison of the four indices of the acceptable points S1, S2, S3 (red), and those of IHC images T1 and T6 (blue); all other images are shown in gray.

At first, we assessed the impact of the differentiation threshold value (Fig 4A). As expected, the percentage of stem cells decreased with higher differentiation thresholds, while Moran’s *I* dropped below the lowest observed experimental values. Entropy increased with differentiation thresholds, reaching acceptable values when the percentage of stem cells was too small. Centrality was mostly insensitive to variations of the differentiation threshold and remained rather constant, around the value 1, therefore highlighting a lack of centrality of the stem cell population. The results showed that in the absence of any spatial rule, no noticeable spatial structure could emerge.

We next tested the impact of spatial signaling according to the following rule: two stem cells, when in contact, signal to each other by increasing the CD133 gene bursting rate. To do so, we set the differentiation threshold value at 0.003, a value obtained without spatial signaling and for which the stem cell percentage, entropy, and centrality index fall within an acceptable range, even though Moran’s *I* remains below the minimum experimental value (Fig 4A). The addition of spatial signaling improved the situation, with a signaling value of 0.3 generating acceptable values for all four indices, although the Moran’s *I* value remained within the low range (Fig 4B). Increasing cell-to-cell signaling intensity increased Moran’s *I*, but pushed the percentage of stem cells index above its maximum limit.

We then assessed the joint effect of varying both the threshold differentiation and the signaling strength. For each (signal,differentiation threshold) pair, we ranked the four indices as either below, within, or above experimental range (Fig 4C–F). We obtained three pairs of parameters acceptable for all four indices: S1 = (0.3, 0.003), S2 = (0.3, 0.004), and S3 = (0.5, 0.004). The corresponding 2D sections are displayed in Fig 4G (S1), 4H (S2), and 4I (S3). The spatial distribution of stem cells is visually close to random, which is consistent with the low value of Moran’s *I* (Fig 4K). In Fig 4J–M, indices associated with S1, S2, and S3 are close to those of IHC images T1 and T6, which represent a case where stem cells are clustered in many small, randomly distributed groups (Fig 2B).

Altogether these results show that the addition of basic spatial rules allows at least some spatial structure to appear, although only the lower range of experimentally observed stem cell clustering can be obtained.

#### Spatial structure and differential cell-cell adhesion.

Cell-to-cell signaling by direct contact might be insufficient to generate the expected spatial clustering. Close examination of simulation movies indeed showed that cell growth and division break stem-to-stem contacts, which results in disrupting stem cell clusters (see an example at https://osf.io/25cy4/files/osfstorage, file Mobile.mov).

To explore how cell movement might affect clustering, we introduced an alternative movement mechanism, called “motile” in Simuscale. Unlike the “mobile” movement above that relies solely on passive forces from daughter cells, motile movement may allow stem cells to arrange their positions more efficiently.

We defined a default value for cell velocity of 0.3 h^−1^, see an example at https://osf.io/25cy4/files/osfstorage (file Motile.mov). We then made velocity contact-dependent: low velocities then mimic cell-to-cell adhesion. We first assumed that both stem-to-stem (S-S) and differentiated-to-differentiated (D-D) cell adhesion would harbour the same value and that stem-to-differentiated (S-D) adhesion could somehow differ.

**Fig 5:**
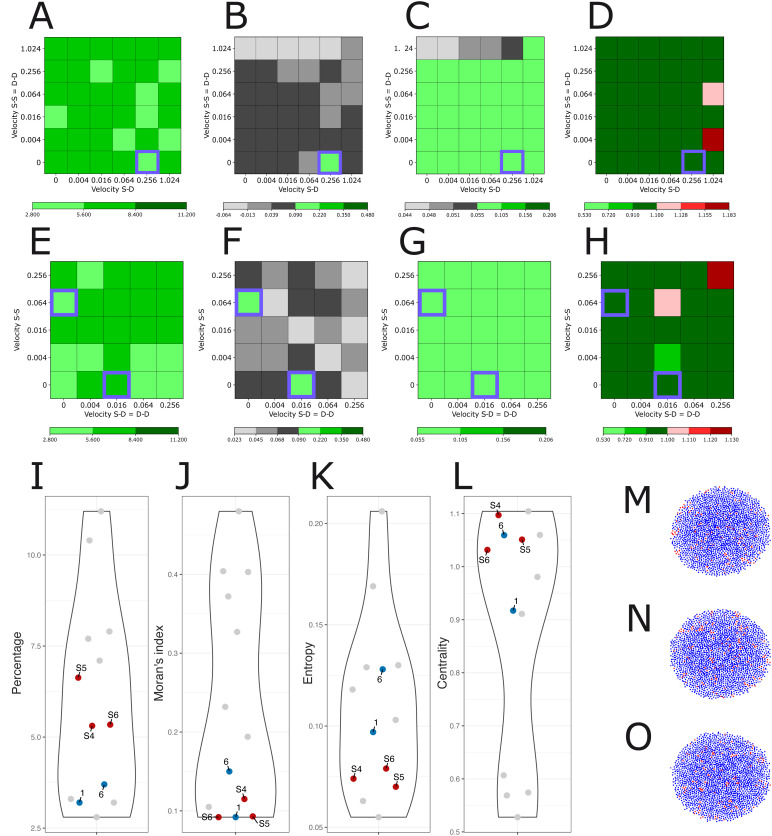
Simulation results for motile cells and differential cell-cell adhesion, while cell-cell contact signaling is reset to 0. A–D. The behavior of our four indices for different values of velocity between stem cells (S-S), equal to the velocity between differentiated cells (D-D) on the Y-axis, and the velocity between stem and differentiated cells (S-D) on the X-axis, with the acceptable points highlighted by violet squares. E–H. The four indices for different values of velocity between stem cells (S-S) on the Y-axis and between stem and differentiated cells (S-D), which is equal to the velocity between differentiated cells (D-D), on the X-axis. I–L. Comparison of the four indices of the acceptable points (red) and those of IHC images T1 and T6 (blue); all other images are shown in gray. M–O. 2D cross-section through the center of the PDT for the acceptable points.

Fig 5A–5D show the values of the four principal indices for different combinations of velocity parameters (S-D, S-S, D-D). A single triplet S4 = (0.256, 0, 0) h^−1^ was found which satisfies all four indices.

We then examined the case where S-D adhesion between stem and differentiated cells equals the D-D adhesion between differentiated cells, whereas the stem-to-stem adhesion might differ. In this case, we obtained two additional acceptable triplets of parameters, S5 = (0.016, 0, 0.016) h^−1^ and S6 = (0, 0.064, 0) h^−1^ (Fig 5E–5H).

We also considered the case in which the adhesion between stem cells (S-S) is equal to the adhesion between stem and differentiated cells (S-D), while the adhesion between differentiated cells (D-D) may differ. However, this scenario does not provide any additional valid points.

Since Moran’s *I* of these three points was quite small, we compared all four indices associated with sections from S4, S5 and S6 with those of the IHC images. We observed that their Moran’s *I* value indeed did not differ significantly from the IHC images T1 and T6 that displayed the lower Moran’s *I* values (see Fig 5I–5L), even though S4 to S6 displayed a higher percentage of CD133^+^ cells.

The 2D cross-sections of the PDT generated from the three configurations, S4, S5, S6, are illustrated in Fig 5M, 5N, and 5O, respectively, showing a similar spatial structure as observed in images T1 and T6 (the one with the lowest Moran’s *I*). However, we also observed that varying the cell adhesion does not significantly impact the spatial distribution of stem cells.

Altogether the addition of differential cell motility only marginally improved the ability of our modeling scheme to reproduce the experimental data.

#### Role of nonlocal diffusive signaling.

**Fig 6:**
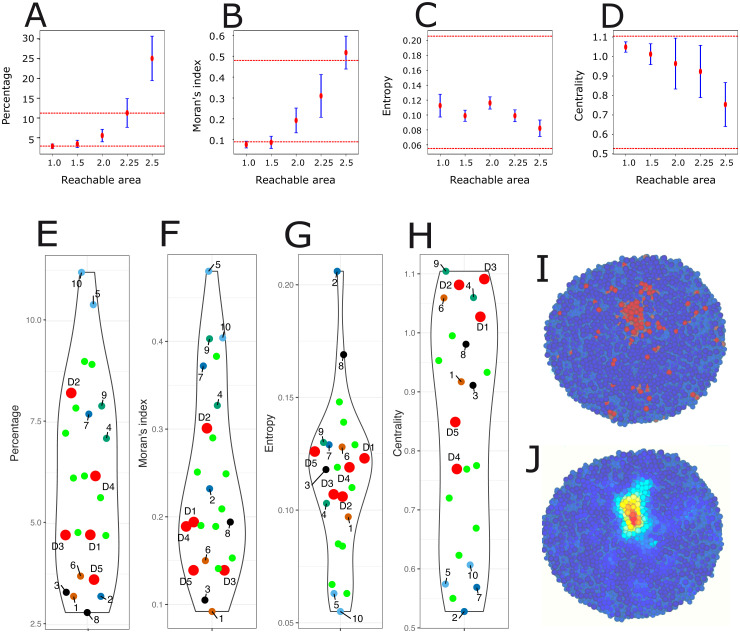
Simulation results with a spatially diffusive signal. A–D. The behavior of the four indices for different values of the reachable area δ. Red points represent the mean over 5 simulations, blue lines the standard deviation, and the two dashed red lines indicate the minimum and maximum experimental values for each index. E–H. Comparison of the values of the four indices between five 2D cross-sections from 5 simulations with reachable area δ=2 (D1 to D5; fluorescent red), 9 successive cross-sections in one simulation (D1; fluorescent green), and the 10 IHC images from Fig 1. I. A 2D cross-section of D1 showing the spatial positioning of stem (red) and differentiated (blue) cells. J. The same 2D cross-section where the intensity of perceived cellular signaling is displayed.

We finally explored a potential role for a nonlocal, spatially diffusing signal, emitted by stem cells only. The effective signaling range (from short to long range) is controled by a so-called *reachable area*, expressed as the parameter δ, see section Signaling. Fig 6 depicts the simulation results when varying the reachable area. These results have been obtained by fixing the signal to 0.3 and the differentiation threshold to 0.004 (S2 in section Spatial structure, differentiation threshold and cell-cell contact signaling).

As expected with a small reachable area δ=1 (a sphere of radius approximately equal to 4.5 µm, akin to a signaling mostly felt in a one cell diameter range), the results were similar to the ones observed with direct cell-to-cell contact signaling. Also expected, when the reachable area was increased to large values (here δ=2.5 being the largest tested value), the percentage of stem cells increased well beyond the maximum value observed in the experimental dataset, together with the Moran’s *I*. Indeed, the nonlocal diffusive signal maintains stem cells in their stem cell state, therefore the farther it is felt the larger the number of cells remaining stem cells.

The most interesting behavior was observed for a short-range reachable area δ=2 (corresponding roughly to a uniform diffusion up to 6.4 µm), where the diffusion signaling can reach up to first-neighbor cells in any given direction. This is the modeling equivalent of a mixture of autocrine, juxtacrine and paracrine signaling, where CSCs can signal both to themselves, to their immediate neighbors and to neighboring cells without direct contact. In this case, we obtained correct values for the four indices, including the highest Moran’s *I* for a correct level in the percentage of stem cells (Fig 6B).

When the simulation-based indices were plotted together with the observed experimental values (Fig 6E–6H), it was clear that the simulations were able to generate quite realistic values, both in average and in their variability. Additionally, a 2D section illustrating the spatial structure of CD133^+^ cells (Fig 6I) demonstrated that the addition of short-range diffusion signal produced the most realistic stem cell spatial distributions by far.

Fig 6J shows how the signal is perceived. It demonstrates that the diffusion signal smoothened out the signaling and allowed to correct the spatial structure by reaching to stem cells that might have been separated by cellular growth.

We reasoned that at least part of the success of the short-range diffusion signal could be due to its effect on de-differentiation events. Those are events where an originally differentiated mother cell would give rise to two stem cell daughters (the level of intracellular CD133 having increased beyond the threshold during the mother lifetime). Such a de-differentiation event is clearly a by-product of our use of a probabilistic GRN and the definition of cell types as the basins of attraction of the GRN model [41]. Calculation of the probability of jumps between cell types has been described in [41]. In the absence of nonlocal diffusion signaling, such de-differentiation events, though rare, occurred in 1.8±0.1% of all cell division events. When nonlocal diffusion signaling was included, de-differentiation occurred at a higher frequency, averaging 3.3±0.5% of all cell division events. This increase could be explained by the increase in stemness associated with the wider spatial distribution of the CD133 signal, which helps stabilize the stem cell population.

We finally also explored the impact of the location of the section made within the simulated PDT. For this we generated 9 successive and evenly distributed (from 10% up to 90% of the structure) cuts through the Z-plane and computed the values for the 4 indices for each section. The results (Fig 6E–6H, green dots) demonstrated that there was a large variability associated to the cutting plane. For some indices, this variability is almost as large as for the 10 experimental sections.

Altogether we demonstrated that short-range spatial diffusion signaling plays a key role in successfully reconstructing the spatial structure of the PDT in this study.

## Discussion

In this work, we developed a multiscale agent-based model to simulate the spatial organization of CD133^+^ cancer stem cells within neuroblastoma patient-derived tumoroids. Our results demonstrated that our in silico multiscale model can reproduce the main expected spatial structure both qualitatively and quantitatively, i.e., stem cells are clustered inside the PDT and surrounded by differentiated cells.

We based our study on the careful quantitative examination of immunohistochemistry images from 5 patient-derived tumoroids grown in vitro. We observed the existence of two mutually exclusive gene expression patterns, with cells expressing either CD133 or SYP. This led to the definition of two phenotypes, CD133^+^ stem cell and SYP^+^ differentiated cell, and to the definition of our GRN built around a toggle switch between those two genes. The percentage of stem cells was much lower than the percentage of differentiated cells, leading to the proposed differential interaction between CD133, SYP and Cyclin E in our GRN. The very definition of two cell types relied upon the experimentally available IHC-based evidence. More complex cell types have been described in the litterature, like noradrenergic (NOR) N-type, mesenchymal (MES) S-type, and intermediate I-type, the very existence of which can be challenged [42–44]. In the present work, we assumed only a stem/progenitor distinction that can be seen as lumping MES and NOR cells into a single category (differentiated cells). We are currently performing joint single-cell RNAseq and spatial transcriptomics using the BMKMANU S3000 platform [45] that should allow us to obtain a much more refined view of the actual complexity of our PDTs.

The extent to which 3D tumoroid cultures recapitulate the known cellular heterogeneity and clinical variability of neuroblastomas [46] makes them especially attractive as preclinical models [47]. Here we document the existence of a quantifiable variability, both inter- and intra-PDTs, and both originating from the same patient-derived xenograft. Although we tried hard to keep culture conditions as constant as possible, in order to minimize purely technical noise, PDT cultures proved to be quite sensitive to technical factors. For example the PDT positioning within the 96-wells plate seems to have some influence on its growth. Regarding intra-PDT variability, the in silico examination of evenly spaced sections along the Z-axis clearly demonstrates that the PDT region that is retained within the cut has a strong influence on the spatial clustering indices. Ideally one would like to obtain similar results from PDTs grown in vitro, i.e., estimate the value of our four indices on many consecutive cuts. Preliminary results suggest that this might be feasible, but with much less control on the positioning of the cut as compared to in silico experiments. Furthermore, technical artefacts during the cut could also limit the extent of section-to-section reproducibility. An elegant alternative would consist in using Light sheet fluorescence microscopy which allows to quickly observe and quantitatively evaluate biological processes and structures in three dimensions with minimal light-induced damage and fading [48]. This should results in 3D images on which we could compute spatial statistics directly comparable to our 3D in silico simulations.

In the present work we did not explore the role of initial seeding conditions. We seeded only a fixed number of stem cells, while in culture the initial cell composition can fluctuate. It could therefore be interesting to explore more diverse initial conditions, by varying both the number and the type of seeded cells. This would allow us to assess how the initial cell composition influences the resulting spatial organization and dynamics of the PDT. The results could then be compared to PDT originating from FACS-sorted stem or differentiated cells or a mix of both.

One remaining question concerns the molecular nature of the diffusive signal. A quick survey of the literature regarding autocrine signaling in cancer stem cells reveals multiple possible candidates, although none were yet identified in neuroblastomas: for example, an autocrine loop using the vascular endothelial growth factor-C (VEGF-C) was described for various cancers [49] or an autocrine loop using the Hepatocyte growth factor (HGF)/c-Met pathway was described for Renal cell carcinoma cancer stem cells [50]. Furthermore, TGF-β has been demonstrated to promote stem cell properties through well documented autocrine loops [51], as well as inducing and maintaining epithelial-mesenchymal transition in transformed human mammary epithelial cells by an autocrine signaling loop, in conjonction with the Wnt signaling pathway [52]. In this last case, the authors have shown that the inhibition of these pathways is sufficient to prevent the acquisition of stem properties by cells.

We could test various ligand-receptor coexpression in our cancer stem cells using single cell transcriptomics data that are currently being generated on our PDT, in order to isolate putative autocrine signaling mechanisms. This could lead to propose a therapeutic strategy in line with [53] that proposed direct targeting of CSCs, using various drugs like AZD9150, a STAT3-targetting agent, or epigenetic drugs such as EZH2 or SETD8 inhibitors to push cancer stem cells out of their stem-like, drug-resistant state, or retinoic acid to force cancer stem cell differentiation.

A number of simplifications in the modeling approach must be acknowledged. No explicit cell death has been considered, since we have no evidence of differential cell death rate between our two cell types and consequently we chose not to use a uniform constant death rate. No explicit biophysical mechanism has been implemented, hence precluding the study of internal mechanical compression known to alter cancer cells behavior (see [54] and references therein). Also, the absence of an explicit extracellular matrix precludes the study of its impact, although matrix stiffness has been shown to be important for the regulation of stemness [55]. Noticeably, a careful examination of our HPS-stained slides showed that very few collagen could be detected in our PDTs. Effects of signaling have not been neglected, but more refined impacts could be implemented, for instance pleiotropic effects—including effect on survival, growth, and migration—are common.

From the experimental point of view, 4D datasets (live images of 3D structures as functions of time) would be ideal but no such dataset is yet available, although recent technological developments make it feasible in the short term [56]. The next best alternative would be longitudinal spatial transcriptomics samples [57].

Despite the above-mentioned simplifications or limitations, our study demonstrates that short-term paracrine signaling is sufficient, in the absence of any other cues, to generate a spatial pattern of CSCs that is qualitatively and quantitatively very close to the one observed experimentally. Furthermore, we found especially interesting that a different study with very different experimental and theoretical approaches reached the very same conclusion as ours, that is that signaling cues provided by neighboring cells play a critical role in shaping the spatial pattern of CSCs [58].

The present framework could be extended to model *bona fide* neuroblastoma tumors. A possible source for experimental evidence could come from recently published spatial transcriptomics studies [43]. One of the main difficulties that can be envisionned is to correctly define the complexity level at which the model should be defined. An inspiration could come from the work of Borau et al. [31] who presented the first multiscale orchestrated framework that integrates molecular, cellular, and tissue-level processes involved in neuroblastoma, using patient-specific data. Major differences with this work would consist in introducing a specific cancer stem cell compartment and in using an explicit dynamical GRN to produce the cell’s molecular content. We are currently working on the inference of such a more realistic GRN using the CARDAMOM inference tool [38]. Such an approach could then lead to generalize the framework to solid tumors when spatial transcriptomics data are available.

## Material, methods and models

### Ethics statement

Frozen cells from orthotopic patient-derived xenograft were obtained from the biorepository of St Jude Children’s Research Hospital (St. Jude Children’s Research Hospital, 262 Danny Thomas Pl, Memphis, TN 38105, USA) at https://www.stjude.org/research/why-st-jude/shared-resources/biorepository.html.

Written consent for each patient from whom tumor cells was used to created OPDX models was obtained by St Jude Children’s Research Hospital as indicated in [59] (https://www.nature.com/articles/nature23647), under the Molecular Analysis of Solid Tumors (MAST) protocol (ClinicalTrials.gov ID NCT01050296, https://clinicaltrials.gov/ct2/show/NCT01050296).

### Generating PDX

Frozen cells from orthotopic patient-derived xenograft (OPDX; [59]) SJNBL013762_X1 were provided by the St. Jude Children’s Research Hospital. This OPDX was derived by grafting a tumor removed from a 15 month-old boy, with adrenal primary tumor location, into the anatomically correct location in the mouse (i.e., the murine adrenal gland). Other features of that tumor included bone marrow metastasis and known molecular alterations consisting in NMYC gene amplification and ALK mutation.

Patient-derived xenograft (PDX) were generated according to [59]: 10^6^ cells were injected subcutaneously (i.e., ectopically) after embedding in 100 µL matrigel (Corning Matrice Matrigel ref 356234) in 6 weeks old NSG-NOD.Cg-Prkdcscid Il2rgtm1Wjl/SzJ (Charles River ref. 614) female mice. The mice were housed in sterilized filter-topped cages and maintained in the P-PAC pathogen-free animal facility (D 69 388 0202; Cancer Research Center of Lyon; CRCL). All animal studies were performed in strict compliance with relevant guidelines validated by the local Animal Ethic Evaluation Committee (C2EA-15) and authorized by the French Ministry of Education and Research (Authorization APAFIS#28836).

### Derivation and culture of neuroblastoma Patient-Derived Tumoroid (PDT)

Fresh tissues from tumor developed in NSG-NOD mice were mechanically minced into small pieces and enzymatic dissociation was done according to the St. Jude Children’s Research Hospital guidelines using accutase for 1-2h at 37°C. 4.10^5^ to 1.10^6^ cells were seeded after embedding in 20 µL matrigel and then placed in 100 µL medium in 96-well ULA plates (BIOFLOAT 96-well U bottom - FaCellitate) in a culture medium based on previously described neuroblastoma tumor-initiating cell lines and PDT derivation protocols [9, 60] which consists of advanced DMEM/F-12 medium (Gibco), 1X B-27 supplement without vitamin A (Gibco), 1X N2 supplement (Gibco), 1x Penicillin-Streptomycin (Gibco), 40 ng/mL hFGF-b (Peprotech), 20 ng/mL hEGF (Peprotech), 10 ng/mL hPDGF-AA (Peprotech), 200 ng/mL hIGF1 (Peprotech) and 10 ng/mL hPDGF-BB (Peprotech).

Cultures were then split without embedding and grown in the same medium and the same 96-well ULA plates. Cultures were supplemented after 7 days by the addition of 50 µL of medium, and then the medium was renewed by half twice a week. PDTs were split every 15–19 days when reaching a diameter of about 1 mm using TrypLE Express Enzyme (ThermoFisher Scientific).

### Histological analyses

Neuroblastoma organoids were fixed and processed as described in [61] when they reached a diameter of 0.8 mm. They were embedded in paraffin (ASP6025, Leica) and processed for histological features by the Research pathology platform East (Anapath Recherche, Cancer Research Center of Lyon (CRCL), Lyon, France). Three µm-thick tissue sections of formalin-fixed, paraffin-embedded tissue were prepared according to conventional procedures. Sections were then stained with hematoxylin, phloxin and safran, and examined with a light microscope.

Immunohistochemistry (IHC) was performed on an automated immunostainer (Ventana Discovery XT, Roche, Meylan, France) using Omnimap DAB Kit according to the manufacturer’s instructions. Slides were deparaffinized at 72 °C using Ventana EZ Prep reagent (Ref: 950-102, Roche) and hydrated, followed by an antigen retrieval method using Ventana Tris-EDTA buffer pH 7.8 (Ref: 950-224, Roche) for 32 mn at 95 °C for CD56 and CD133 and for 60 mn for Synaptophysine at 95 °C.

Sections were incubated with:

- a mouse anti-CD56 (Novocastra, NCL-L-CD56-504) diluted at 1:100 for 32 mn. Sections were then sequentially incubated with a rabbit anti-mouse IgG2b (Abcam, ab125907) for 32 mn and then an anti-rabbit-HRP (Roche, 760-4311) for 16 mn
- a rabbit anti-CD133 (Cell signaling Technology, 86781) diluted at 1:500 for 32 minutes then an anti-rabbit-HRP (Roche, 760-4311) was applied on sections for 16 mn.
- a mouse anti-Synaptophysin (Novocastra, NCL-L-SYNAP-299) diluted at 1:200 for 32 mn. Sections were then sequentially incubated with a rabbit anti-mouse IgG1 (Abcam, ab133469) for 32 mn and then an anti-rabbit-HRP (Roche, 760-4311) for 16 mn.
- anti-Phox2B (Fine test, FNab06409) diluted at 1:200 for 20 mn on the autostainer Dako.

Staining was visualized with 3,3’-diaminobenzidine as a chromogenic substrate for 8 mn. The sections were counterstained with Gill’s Hematoxylin (Ref: 760-2021, Roche) for 8 mn and post counterstained with Bluing reagent (Ref: 760-2037, Roche) for 4 mn. The slides were washed in warm tap water with detergent and dehydrated in graded ethanol and methylcyclohexane, then coversliped in permanent mounting media Pertex (Ref: 00801-FR, HistoLab). Finally, sections were scanned with panoramic scan II (3D Histech, Budapest, Hungary) at 20X.

Five PDT from the same culture were prepared and processed for IHC. For each PDT, we analyzed two sections along the Z-axis, resulting in 10 immunohistochemistry images, numbered T1 to T10. Each pair of images (T1,T6), (T2,T7), (T3,T8), (T4,T9), and (T5,T10) comes from the same PDT. All images are available in S1 File.

We checked for the expression of CD133, a known stemness marker [53, 62–64] shown to suppress neuroblastoma differentiation [65] via the inhibition of RET, a tyrosine kinase receptor involved in neuroblastoma differentiation [66]. This allowed us to define two cell types: stem cells, expressing CD133, and differentiated cells expressing synaptophysin (SYP) (see section Spatial distribution of stem cells). It is important to stress that throughout this work, we will consider cell types as the basins of attraction of the GRN model [41]. The basin of attraction is a notion related to deterministic dynamics, consequently in the current framework where we consider a stochastic model of gene dynamics it refers to the basin of attraction of the coarse approximation, in the form of a system of ordinary differential equations, of a piecewise-deterministic Markov process [41].

IHC data is available at https://osf.io/e6vug/files/osfstorage.

### Image analysis

Images were open and analyzed using *QuPath* [67], an open source software for digital pathology image analysis.

In order to accurately identify cells in the IHC images, some parameters were modified from the default configuration when detecting positively stained cells for CD133 (respectively, SYP). We took into account the ‘background radius’, i.e., the size of the region around each cell nucleus used to estimate the local background intensity, which was set to 9 µm (resp., 6 µm).

Nuclei must have a mean intensity greater than a threshold, called intensity threshold and set at 0.05 for both markers, to be detected. Detected nuclei with an area less than a minimum, set to 2 µm for both markers, were discarded.

The mean cell count was 2,548 ± 336 cells per section. The mean cell radius was 4.5 ±0.3 µm.

### Statistical methods

To analyze the spatial distribution of stem cells in IHC images of our PDT we focused on the following statistical indices: their percentage, Moran’s index, entropy, and centrality.

**Percentage**: It is calculated as 100 times the ratio of the number of CD133^+^ cells to the total number of cells.

**Moran’s index**: Moran’s index (thereafter named Moran’s *I*) is a statistical indicator of spatial autocorrelation, meaning it quantifies how similar or clustered values are based on their spatial locations [68]. Stem cell Moran’s *I* is computed as follows


$$I=N\;\frac{\sum_{i,j=1}^{N}w_{i,j}(x_{i}-\bar{x})(x_{j}-\bar{x})}{\sum_{i,j=1}^{N}w_{i,j}\sum_{i=1}^{N}(x_{i}-\bar{x}{)}^{2}},$$

where *N* is the total number of cells, $(x_{i}{)}_{i=1,\ldots ,N}$ is the observation data (represented by a binary vector where *x*_*i*_ = 0 for differentiated cells and *x*_*i*_ = 1 for stem cells), $\bar{x}$ its mean, and {*w*_*i*,*j*_} is the neighborhood weight matrix, with *w*_*i*,*i*_ = 0 for all *i* and $\sum_{j=1}^{N}w_{i,j}=1$. Moran’s *I* ranges between −1 and 1. If Moran’s *I* value is close to 0, the distribution of stem cells is random. If I=−1, the distribution is perfectly dispersed, whereas if *I* = 1 stem cells are perfectly clustered. A high Moran’s *I* indicates that stem cells tend to cluster together.

In practice, two cells are considered to be neighbors if their distance is less than or equal to 15 µm, which is in agreement with observations across all 10 IHC images.

**Entropy**: The entropy index, usually denoted by *H*, characterizes the distribution of distances between cells. The entropy index for stem cells is given by [69]


$$H=-\sum_{i\in J}p_{i}\;log(p_{i}),$$

where *p*_*i*_ represents the probability that the distance between stem cells (CD133^+^) falls within bin *i* of the distance histogram, *J* being the set of all bins. This index provides complementary information about stem cell clustering.

**Centrality**: The centrality index is the ratio between the mean distance of all stem cells to the center of the IHC image of the PDT and the mean distance of all differentiated cells to the same center.

In addition, to better understand the spatial dispersion of stem cells, we computed the *intra-coefficient* $a_{SS}$ introduced by Jensen and Michel [39], which quantifies the spatial dependency between cells of the same type, here the stem cell type denoted by S. More precisely,


$$a_{SS}=\frac{N_{t}-1}{N_{S}(N_{S}-1)}\;\sum_{i=1}^{N_{S}}\frac{N_{S}(C_{i},r)}{N_{t}(C_{i},r)},$$

where *N*_*S*_ is the cell count of type S, *N*_*t*_ is the total number of cells, and $N_{S}(C_{i},r)$ (resp. $N_{t}(C_{i})$) is the number of type S cells (resp. all types) in the neighborhood of radius *r* around stem cell *C*_*i*_ (without counting *C*_*i*_). The fraction 0/0 is considered equal to 1 in the right side. For all *r* > 0, the average over all cells of type *S* of this coefficient equals 1.

In [39], the analytical form of the variance of the intra-coefficient has been computed under the null hypothesis that no spatial correlation exist. Under the null hypothesis [39],


$$P\left(|a_{SS}-1|\leq q_{\alpha }^{SS}\right)\geq 1-\alpha ,$$

where α is the level of confidence and $q_{\alpha }^{SS}=\sqrt{\sigma^{2}(a_{SS})/\alpha }.$

In section Variance statistics for spatial dispersion of stem cells, we consider α=0.05 and compute the intra-coefficient of the IHC images along with its confidence interval $\left[1-q_{\alpha }^{SS},1+q_{\alpha }^{SS}\right]$, i.e., a confidence level of at least 95% under the null hypothesis. If the observed value of the intra-coefficient is greater than 1, we deduce that S-type (stem) cells tend to aggregate. Furthermore, if $a_{SS}$ lies outside the confidence interval, i.e., $|a_{SS}-1|>q_{\alpha }^{SS}$, we can reject the null hypothesis.

### Multiscale modeling framework

Simuscale [34] is a multiscale individual-based modeling platform for performing numerical simulations of heterogeneous populations of individual cells. It is intended for cells that experience temporal evolution (movement, differentiation, death, division) and accounts for physical and biochemical cell interactions. A Simuscale model is a priori described at the cellular and the population level. The cellular level describes the dynamics of single cells, and allows various formalism. The internal cell state includes, in this work, cell size and position, mRNA and protein levels for each gene (see section Gene regulatory networks). At the population level, cells evolve in a bounded 3D domain, they can divide (and generate two daughter cells), differentiate, or move (see section Cell motion). Spatially, cells are represented as visco-elastic spheres with a rigid core, and have two radii: the radius of the core, named ‘internal radius’, and the radius of the sphere, named ‘external radius’.

Simuscale implements the physical simulator that manages the simulations at the population level. Biochemical interactions occur between cells that are in contact with each other, through intercellular signals that can be known to all or to a subset of the cells only. Simuscale runs a simulation over a specified time interval, updating the cell population at given time steps, and it generates an output file containing the state of each cell at each time step, and the tree of cell divisions (and deaths when cells die).

Pseudo-code and a flow chart summarizing Simuscale architecture are available in S1 File.

### Gene regulatory networks

To build the multiscale model of neuroblastoma PDT, we first introduce a gene regulatory network (GRN), comprising the three genes CD133, SYP and Cyclin E. This GRN governs cell fate decisions through a toggle switch between CD133 and SYP, as described in section Cell fate. Cyclin E, a key regulator of the G1/S phase transition [70], controls the entry into proliferation. Mathematically, the GRN is written as the bursty model, a piecewise deterministic Markov process which couples deterministic and stochastic processes in each gene i∈G:={CD133,SYP,CyclinE} to produce mRNA (*M*_*i*_) and proteins (*P*_*i*_) (see [37, 71]). Furthermore, this GRN is implemented in Simuscale, as in [35].

More explicitly, the dynamics of mRNA and protein concentrations in a gene, $\left(M_{i}(t),P_{i}(t)\right)$ for each i∈G, evolve deterministically with


$$\left\{ \begin{array}{c} M_{i}^{\prime }=-d_{0,i}M_{i},\\ P_{i}^{\prime }=s_{1,i}M_{i}-d_{1,i}P_{i}, \end{array}\right.$$

until gene *i* experiences a burst, where *s*_1,*i*_ is the protein synthesis rate of gene *i*, and *d*_0,*i*_ is the mRNA degradation rate, which is much larger than *d*_1,*i*_, the protein degradation rate. A burst causes the quantity of mRNA (*M*_*i*_) to increase by a random, discontinuous jump, sampled from an exponential distribution.

We fix the degradation rate of mRNA equal to 1 h^−1^ for all genes, i.e., *d*_0,*i*_ = 1 h^−1^ for all gene i∈G, the protein degradation rate *d*_1,CD133_ = 0.001 h^−1^, $d_{1,\text{SYP}}=d_{1,\text{P}}=0.01$ h^−1^, and $s_{1,i}=0.01\;d_{1,i}$ h^−1^ for all gene i∈G.

Additionally, the burst rate of each gene *i* is defined as follows


$$k_{on,i}^{\theta }(P)=k_{0,i}+(k_{1,i}-k_{0,i}){\left(1+\exp\left(-\sigma_{i}^{\theta }(P)\right)\right)}^{-1},$$

where *P* is the vector of proteins concentrations, *k*_0,*i*_ and *k*_1,*i*_ correspond to the minimum and maximum burst frequencies of gene *i*. Three scenarios are considered in this study for integrating cell-cell signaling within the burst frequency of the CD133 gene (noticeably, only CD133 gene dynamics are impacted by signaling in this work):

**1.** No signaling,


$$\sigma_{i}^{\theta }(P)=\beta_{i}+\sum_{j\in G}\theta_{ij}P_{j},$$

where $\beta_{i}$ represents the basal activity of gene *i*, with $\beta_{\text{CD133}}=-3$, $\beta_{\text{SYP}}=\beta_{\text{CyclinE}}=-5$ (no unit). Values $\{\theta_{ij}{\}}_{i,j\in G}$ denote the influence of gene *i* onto gene *j*, as encoded in the gene-gene interaction matrix between the gene protein concentrations, with their interaction strengths (no unit) specified in Table 1.

**2.** Stem cell - stem cell contact signaling,


$$\sigma_{i}^{\theta }(P)=\beta_{i}+\sum_{j\in G}\theta_{ij}P_{j}+\gamma S,$$

where S∈{0,1} is the signaling-by-contact state and γ>0 is the signaling strength, which will be examined in section Spatial structure, differentiation threshold and cell-cell contact signaling.

**3.** Stem cell-driven diffusion signaling,


$$\sigma_{i}^{\theta }(P)=\beta_{i}+\sum_{j\in G}\theta_{ij}P_{j}+S_{\text{diff}},$$

where *S*_diff_ represents the intensity of the diffusion signal perceived by a stem cell as defined in section Signaling.

### Signaling

Cell-cell contact signaling is implemented as in [35]. Stem cell signaling to other stem cells is considered in section Spatial structure, differentiation threshold and cell-cell contact signaling, and follows this rule: two stem cells can communicate only if they are in contact (the distance of their centers is smaller than the sum of their external radii).

Long-range signaling via spatially diffusing signals *S*_diff_ has recently been implemented in Simuscale. Instead of implementing, for instance, the three-dimensional screened Poisson equation, we assume that signals emitted by a cell (here, only a stem cell) diffuse according to a Gaussian density with variance δ/2, centered in a point *h* inside the cell (typically, the center of the cell). In a population of *N* cells, the total signal *S*_diff_ perceived by a cell at position *l* is the Gauss transform of *N* signal sources *l*ocated at positions *h*_*j*_, with strengths *q*_*j*_,


$$S_{\text{diff}}=\sum_{j=1}^{N}q_{j}\;.\;e^{\left(-\frac{||l-h_{j}|{|}^{2}}{\delta }\right)},$$

Classical direct numerical methods would require the evaluation of *M*_*S*_ sources at *M*_*T*_ targets (with *M*_*S*_ denoting the number of secreting cells and *M*_*T*_ the number of receptive cells) at each time step, which would be computationally infeasible for the number of cells considered here. Instead we rely on the fast Gauss transform algorithm introduced by Greengard and Strain [72]. All thresholds are chosen such that the relative error at any target remains below a user-specified tolerance ε (here, ε=0.01). We fix *q*_*j*_ = 0.3 for the intensity of each stem cell signaling, and values of δ, which we call the *reachable area* (and is sometimes referred to as the diffusion length squared), will be studied in section Role of nonlocal diffusive signaling. A movie of the diffusion signaling is available at: https://osf.io/25cy4/files/osfstorage (Diffusion_coef_2.mov). A flow chart summarizing the implementation of diffusion in Simuscale is available in S1 File.

Whatever the nature of the signal (cell-cell contact or spatially diffusing), it is assumed to impact only the CD133 gene dynamics through its burst rate.

### Cell motion

For most scenarios considered in this work, only “mobile” movement is used for all cells [34]: in this case, cells move only because forces are applied on them, coming either from the growth of the mother cells or from daughter cells generated during cell division.

In some scenarios, “motile” cell movement (as described in [35]) is used. In this case, cells move by themselves, and we considered random cell motion within a 3D domain, where the initial velocity of each cell is set to 0.3 h^−1^.

When a cell encounters another cell, its velocity changes to a predefined velocity: when a stem cell encounters another stem cell, we call it a S-S velocity, when a stem cell encounters a differentiated cell (or conversely) we call it a S-D velocity, and when a differentiated cell encounters a differentiated cell we name it D-D velocity. Notably, if a stem cell simultaneously contacts multiple cell types and at least one of its neighbors is a stem cell, its velocity is set to the S-S velocity value.

### Cell decision making

Contrary to *in vivo* tumors (see, e.g., [73]), PDT do not display a necrotic core, since gas and nutrients do flow freely through the relatively small structure. Therefore for simplification purposes, no cell death was implemented in the current version of the model.

Cell growth is a continuous linear process where cells augment their volume, which may vary from a minimum volume set to 1 to a maximum volume set to 2.2. From *V*_birth_ = 1 (daughter cell volume at birth, radius r≈0.62 spatial units, that is r≈4 µm), or $V_{\text{birth}}\geq 1$, the volume increases up to $V_{\text{division}}\geq 2$ (r≈5 µm), where cells are ready to divide, pending upon their internal state values (see section Cell fate). Each daughter cell inherits half the volume of the mother cell at birth, hence *V*_birth_ can be larger than 1. The conversion of a spatial domain unit in µm is based on a computed mean cell radius *r* = 0.7 corresponding to an experimentally average radius *r* = 4.5 µm.

Differentiation is also based on the cell’s internal content (see section Cell fate). Two cell types are considered: stem cells (CD133^+^) and differentiated cells (SYP^+^). Once the cell type is acquired, some properties are directly associated to it (for example both signaling emission and perception are a stem cell characteristic) whereas other emerge from the behavior of the underlying GRN (for example, differentiated cells proliferate faster due to the strong positive influence of the SYP protein on the burst frequency of the Cyclin E gene, see section Cell fate for details).

### Initial conditions

Each simulation is initialized with 15 stem cells (CD133^+^ cells) gathered side by side at the center of a 3D cube with edge length of 80 units. The spatial domain unit corresponds to 6.4 µm, so each length of the cube is 512 µm.

Molecular contents of the gene regulatory network (GRN) are initially set as follows:

- The CD133 protein value is set to twice the differentiation threshold (see section Cell fate) value in all cells, ensuring that the first generation of daughter cells can remain stem cells. All other protein values are initialized to 0 molecule.
- mRNA values of all genes are set to 0 molecule, except for CD133, for which each cell receives an initial random value computed from an exponential burst distribution with a mean of 50 molecules.

### Simulations

We performed 10 repetitions with the same parameter set but using different seeds, except for the diffusion study, where we performed only 5 repetitions due to computing time (calculation time exceeding 10 hours per run). All simulations were stopped when the total number of cells exceeded 50,000. This corresponds to the average cell counts from PDT in culture when they were split, i.e., when their size approached 1 mm in diameter (see section Derivation and culture of neuroblastoma Patient-Derived Tumoroid (PDT)). Interestingly, when cutting through the 3D structure, we obtained the same cell count (between 2,500 and 3,000) as observed in the IHC images (see section Image analysis).

The computational approach is lattice-free; cells boundary conditions are reflexive (cells cannot cross the boundaries) and free-flux boundary conditions have been implemented for diffusive signals. The time step of all simulations is set to *dt* = 0.1 hour.

The resolution of the stochastic GRN model proceeds as follows: on a time interval [*t*, *t* + *dt*], the time of the next burst (for each gene) is computed based on the updated burst rate *k*_*on*,*i*_. If a burst occurs in the interval [*t*, *t* + *dt*], then the model is solved up to the burst time, then mRNA quantity (*M*_*i*_) is increased by a random, discontinuous jump, sampled from an exponential distribution with a mean of 50 molecules, and the simulation run resumes, either up to the next time of burst or up to *t* + *dt*. If no burst occurs then the deterministic system is solved on the time interval [*t*, *t* + *dt*].

All codes are available at https://github.com/ThiNhuThaoNGUYEN/SimusNeuroblas.

## Supporting information

S1 Fig IHC staining using clinical markers routinely used for NB diagnosis. Shown is the original tumor (image courtesy of the St Jude Hospital, first line), our own derived PDX (second line), and the corresponding derived PDT (third line) at passage 10. Positive cells are stained in brown.
(PDF)

S1 File Supporting information. This file contains Simuscale pseudo-code, flowchart, and Diffusive signaling flowchart. It also contains all IHC images T1 to T10.
(PDF)
