# Supplementary figures and images for: Multiscale modeling of the spatial structure of stem cells in neuroblastoma patient-derived tumoroids reveals a critical role for a short-range diffusive process
Source: PLoS Comput Biol. 2026 Mar 31;22(3):e1014137. doi: 10.1371/journal.pcbi.1014137 (PMC13061327; doi:10.1371/journal.pcbi.1014137)

Synaptophysin

CD56

PHOX2B

Patient

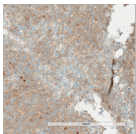

PDX

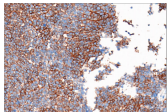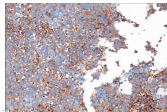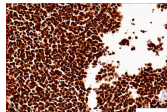

PDTs

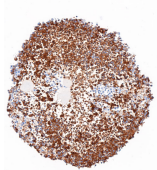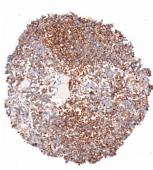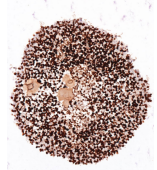

100  $\mu$ m

Supplement: S1 Fig — Shown is the original tumor (image courtesy of the St Jude Hospital, first line), our own derived PDX (second line), and the corresponding derived PDT (third line) at passage 10. Positive cells are stained in brown. (PDF) [file pcbi.1014137.s001.pdf]
