# Supplementary material for: Multiscale modeling of the spatial structure of stem cells in neuroblastoma patient-derived tumoroids reveals a critical role for a short-range diffusive process
Source: PLoS Comput Biol. 2026 Mar 31;22(3):e1014137. doi: 10.1371/journal.pcbi.1014137 (PMC13061327; doi:10.1371/journal.pcbi.1014137)

Multiscale modeling of the spatial structure of stem cells in neuroblastoma patient-derived tumoroids reveals a critical role for a short-range diffusive process

Supporting File

Thi Nhu Thao Nguyen, Catherine Koering, Elodie Vallin, Sandrine Gonin-Giraud, Laura Broutier, Samuel Bernard, Fabien Crauste, Olivier Gandrillon

Simuscale Flowchart

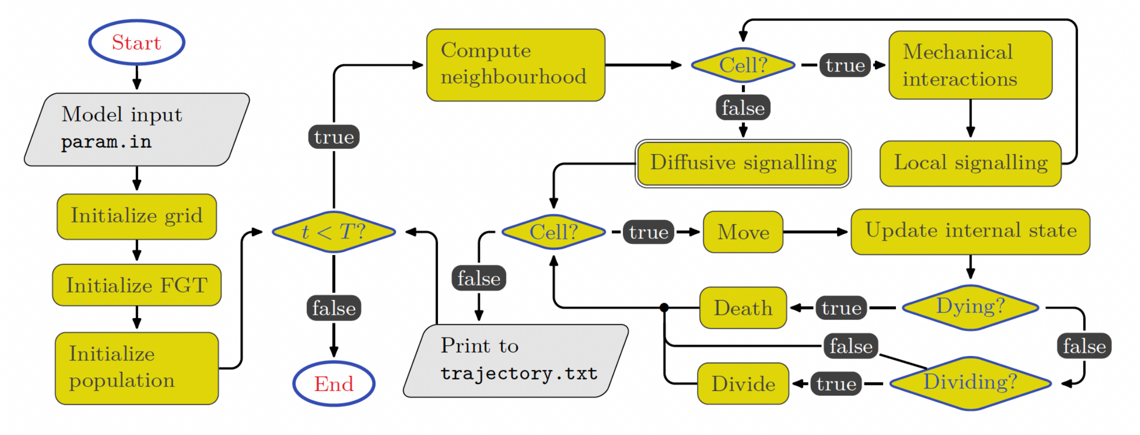

Simuscale: Diffusive signalling flowchart

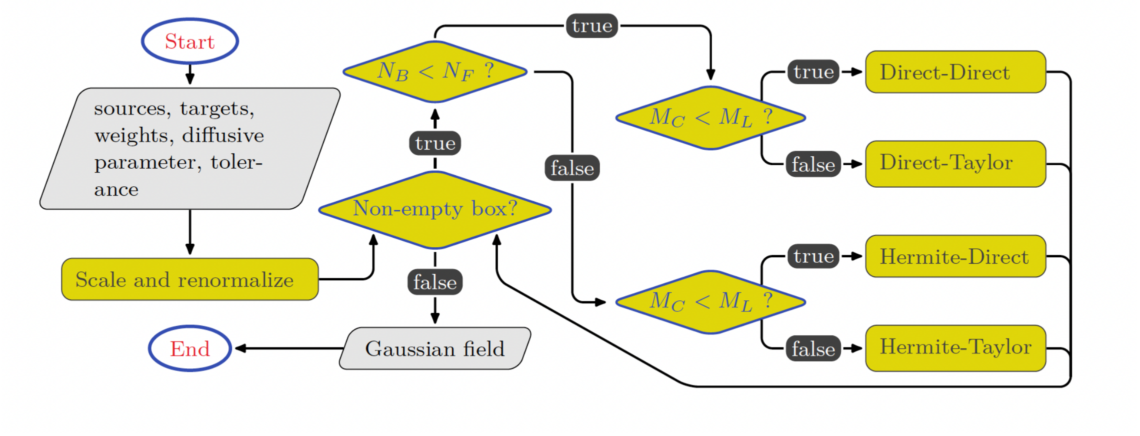

Simuscale Pseudocode

```
Read("param.in");                          // Tfinal, dt, signal lists, etc
InitializeGrid();                          // local interaction management
InitializeFGT();                           // diffusive interaction management
InitializePopulation();                    // population
t = 0;

while (t < Tfinal) {                       // main loop
  findNeighbourhood();                     // find neighbouring cells (single loop)
  for (cell : population) {                // compute mechanical interactions
                                           // and local signalling
    for (neighbour : neighbourList) {      // accumulate mechanical forces
                                           // from neighbours
        cell->ComputeMechanicalForce(neighbour);
    }
    for(signal : localSignalList) {        // accumulate signals from neighbours
        cell->ComputeLocalInteraction(signal);
    }
  }
  for(signal : diffusiveSignalList) {      // compute diffusive field
    ComputeDiffusiveInteraction(signal);   // fast-Gauss-transform (single loops)
  }
  for(cell : population) {                 // update with time step dt
    cell->Move(dt);                        // move
    cell->InternalUpdate(dt);              // update internal state, fate
    if(cell->isDead) {                     // process dying cell
      population->Remove(cell);            // remove cell when marked as dead
      continue;                            // dead cells cannot divide
    }
    if(cell->isDividing) {                 // apply division rules
      daughterCell = cell->Divide();       // create new cell
      population->AddCell(daughterCell);   // add daughterCells to population
    }
  }
  PrintState("trajectory.txt");
  t += dt;
}

Finalize();                                // print final output, clean up
```

Immunohistochemistry Images

Five Patient-Derived Tumoroids (PDT) from the same culture were prepared and processed for immunohistochemistry. For each PDT, two sections along the Z-axis were analyzed, resulting in 10 IHC images, numbered T1 to T10. Each pair of images (T1,T6), (T2,T7), (T3,T8), (T4,T9), and (T5,T10) comes from the same PDT. These images are displayed on the next pages.

Sections T1 (top) and T6 (bottom) of the same PDT.

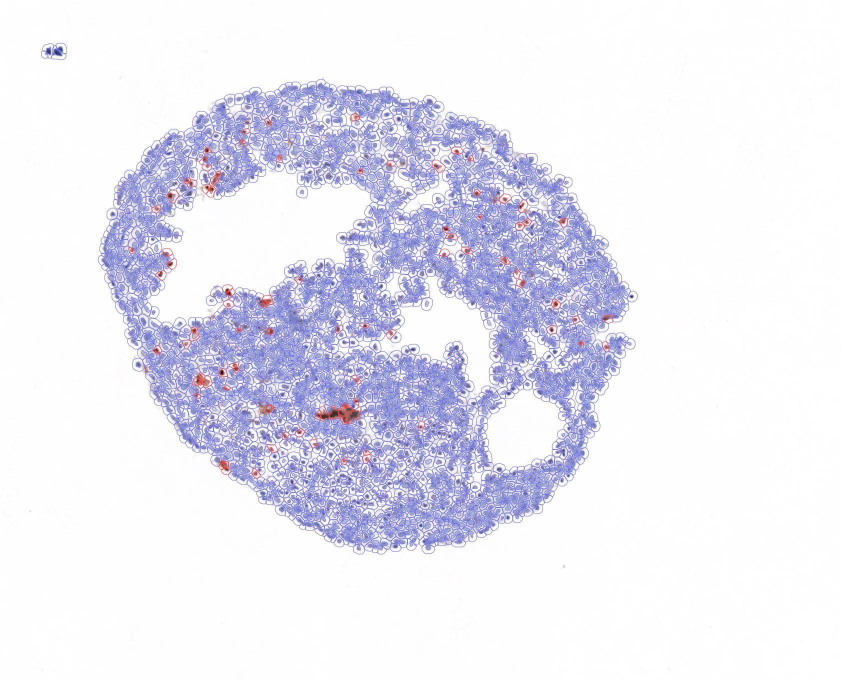

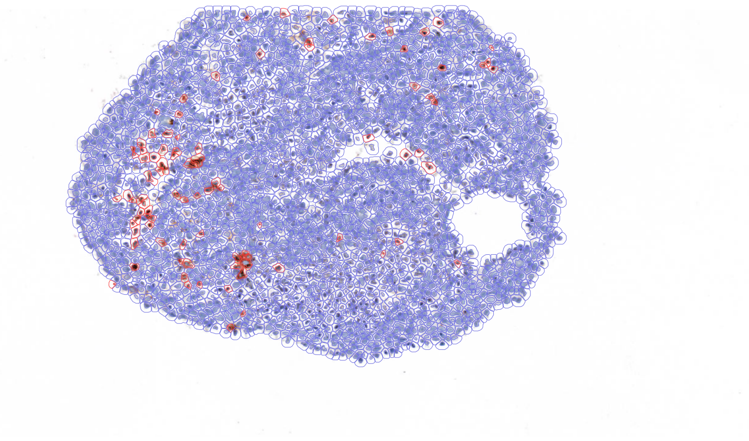

Sections T2 (top) and T7 (bottom) of the same PDT.

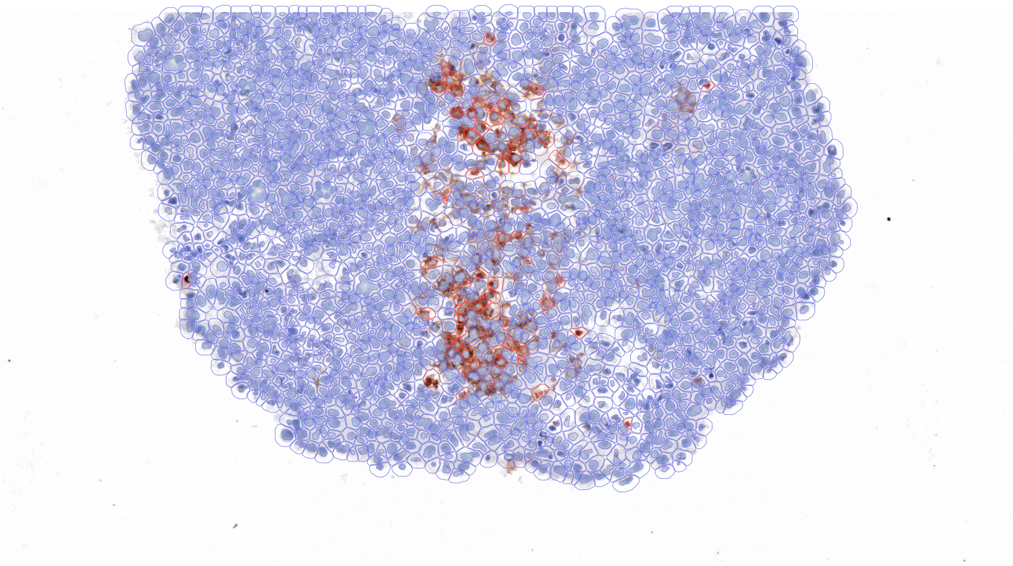

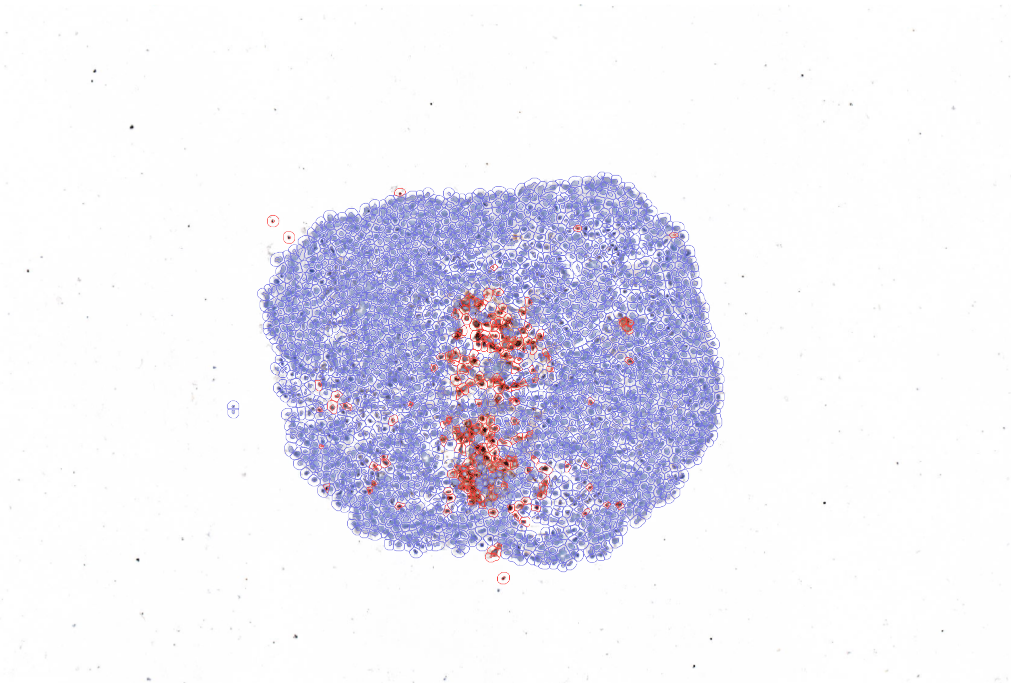

Sections T3 (top) and T8 (bottom) of the same PDT.

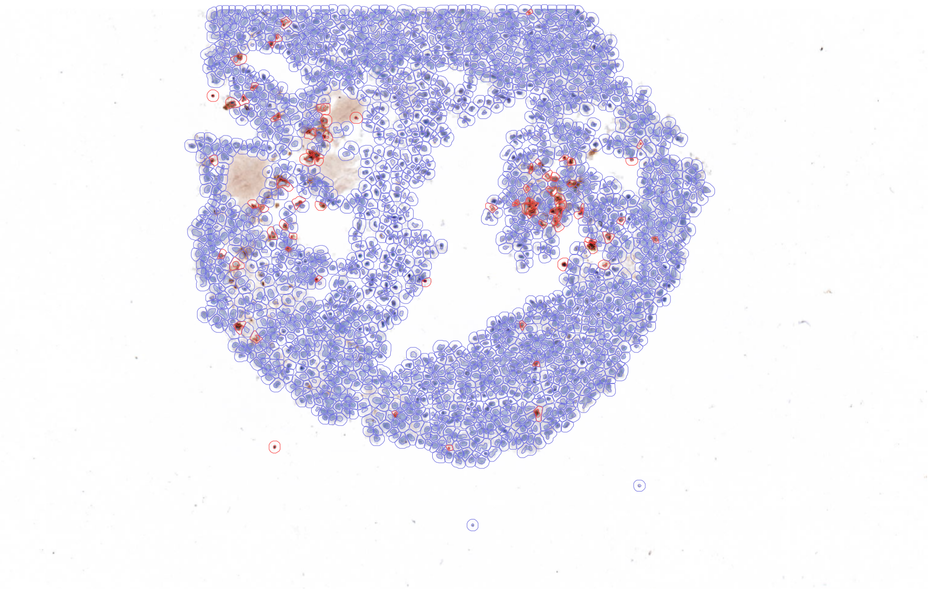

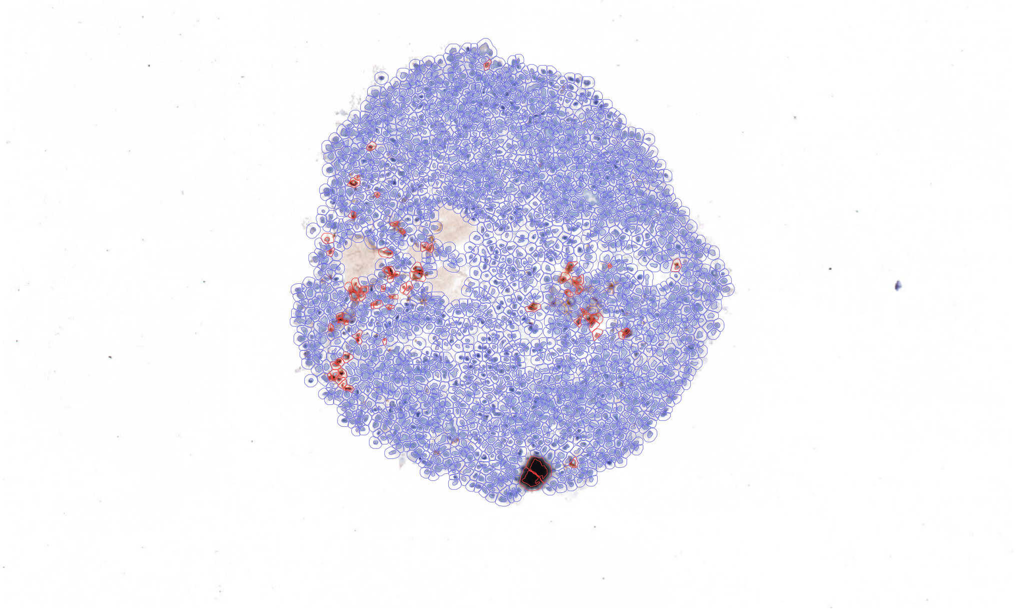

Sections T4 (top) and T9 (bottom) of the same PDT.

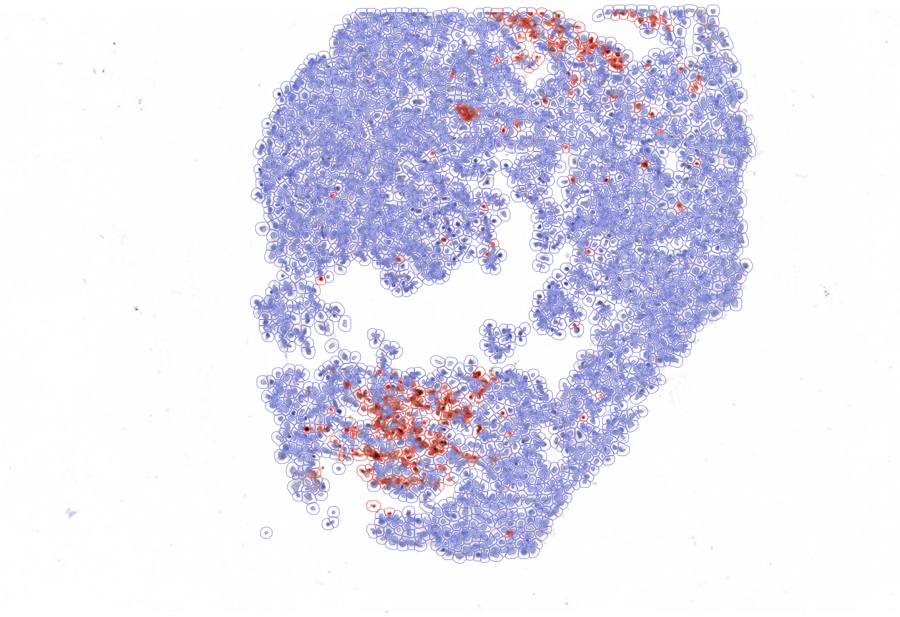

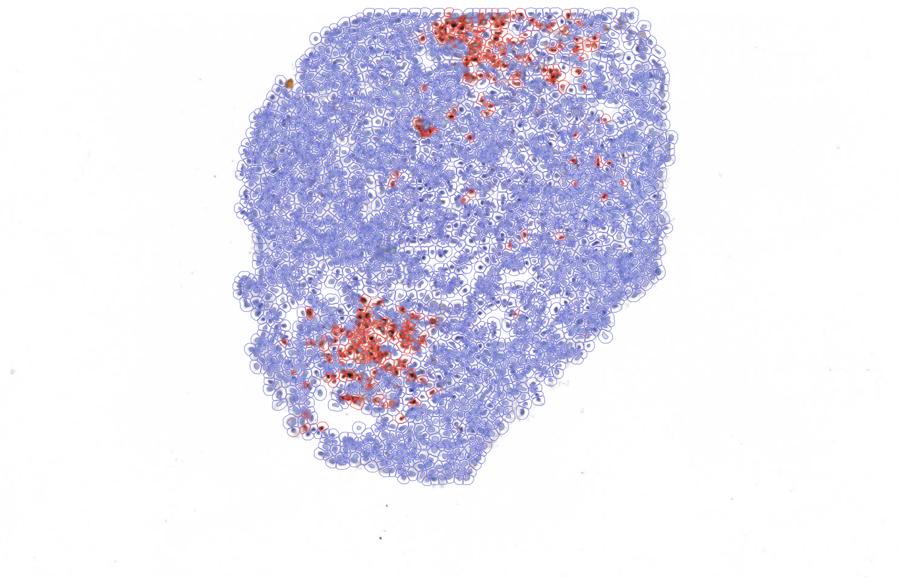

Sections T5 (top) and T10 (bottom) of the same PDT.

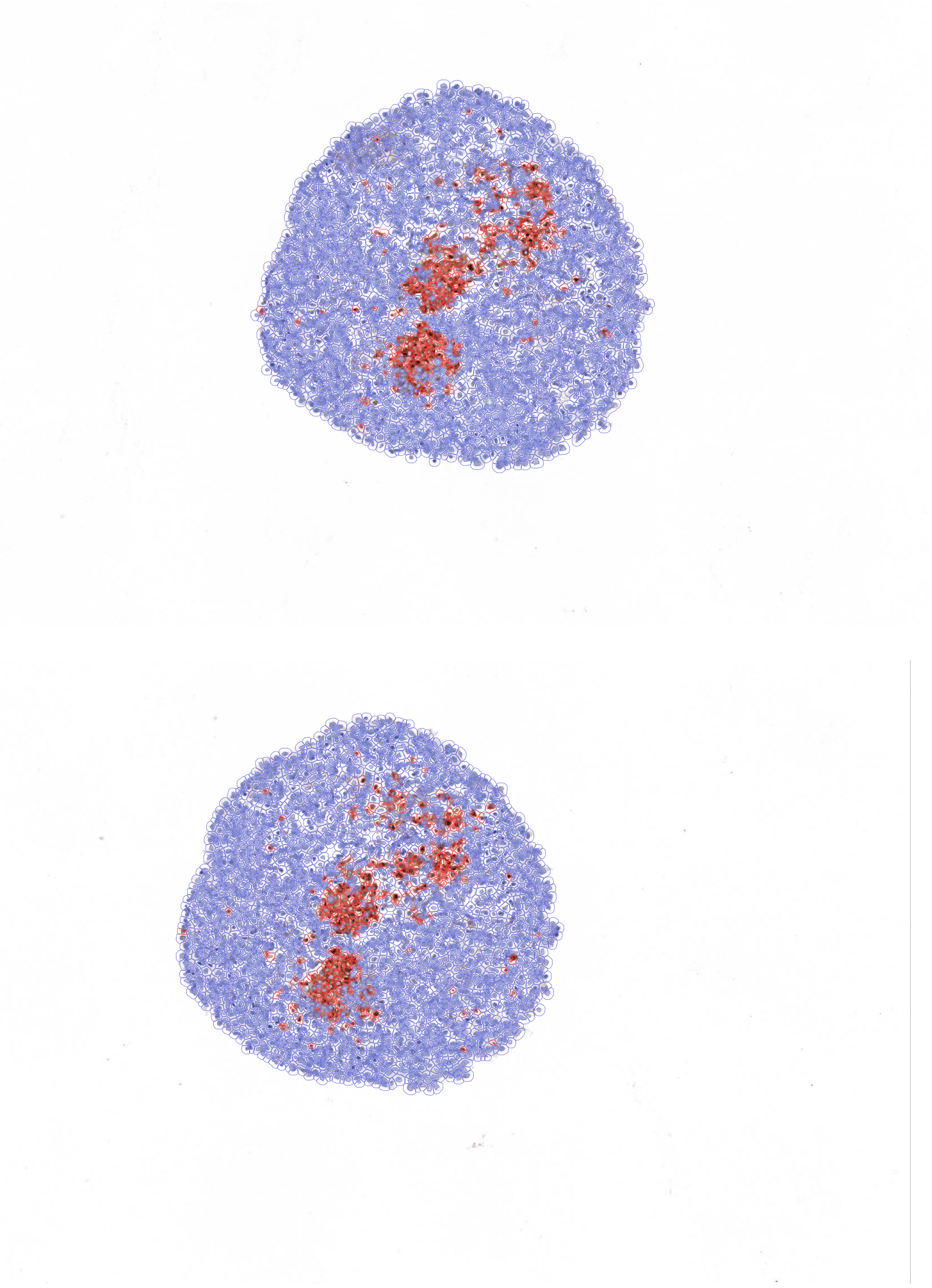

Supplement: S1 File — This file contains Simuscale pseudo-code, flowchart, and Diffusive signaling flowchart. It also contains all IHC images T1 to T10. (PDF) [file pcbi.1014137.s002.pdf]
